# Detection and Quantification of Tomato Paste Adulteration Using Conventional and Rapid Analytical Methods

**DOI:** 10.3390/s20216059

**Published:** 2020-10-24

**Authors:** Flora Vitalis, John-Lewis Zinia Zaukuu, Zsanett Bodor, Balkis Aouadi, Géza Hitka, Timea Kaszab, Viktoria Zsom-Muha, Zoltan Gillay, Zoltan Kovacs

**Affiliations:** 1 Institute of Bioengineering and Process Control, Department of Measurements and Process Control, Faculty of Food Science, Szent István University, 1118 Budapest, Hungary; vitalis.flora@hallgato.uni-szie.hu (F.V.); zaukuu.john-lewis.zinia@hallgato.uni-szie.hu (J.-L.Z.Z.); bodor.zsanett@hallgato.uni-szie.hu (Z.B.); aouadi.balkis@hallgato.uni-szie.hu (B.A.); kaszab.timea@szie.hu (T.K.); zsomne.muha.viktoria@szie.hu (V.Z.-M.); gillay.zoltan@szie.hu (Z.G.); 2 Institute of Food Technology, Department of Postharvest, Commercial and Sensory Science, Faculty of Food Science, Szent István University, 1118 Budapest, Hungary; hitka.geza@szie.hu

**Keywords:** food adulteration, authentication, tomato paste, soluble solid content, Bostwick consistency, NIR spectroscopy, electronic tongue, chemometrics

## Abstract

Tomato, and its concentrate are important food ingredients with outstanding gastronomic and industrial importance due to their unique organoleptic, dietary, and compositional properties. Various forms of food adulteration are often suspected in the different tomato-based products causing major economic and sometimes even health problems for the farmers, food industry and consumers. Near infrared (NIR) spectroscopy and electronic tongue (e-tongue) have been lauded as advanced, high sensitivity techniques for quality control. The aim of the present research was to detect and predict relatively low concentration of adulterants, such as paprika seed and corn starch (0.5, 1, 2, 5, 10%), sucrose and salt (0.5, 1, 2, 5%), in tomato paste using conventional (soluble solid content, consistency) and advanced analytical techniques (NIR spectroscopy, e-tongue). The results obtained with the conventional methods were analyzed with univariate statistics (ANOVA), while the data obtained with advanced analytical methods were analyzed with multivariate methods (Principal component analysis (PCA), linear discriminant analysis (LDA), partial least squares regression (PLSR). The conventional methods were only able to detect adulteration at higher concentrations (5–10%). For NIRS and e-tongue, good accuracies were obtained, even in identifying minimal adulterant concentrations (0.5%). Comparatively, NIR spectroscopy proved to be easier to implement and more accurate during our evaluations, when the adulterant contents were estimated with R^2^ above 0.96 and root mean square error (RMSE) below 1%.

## 1. Introduction

Tomato is one of the most popular and important crops in the world [1]. Its worldwide production was about 188 million tons in 2018, but a dynamic consumption growth observed in China and India has led to an even higher global demand [2,3]. The favorable organoleptic properties of tomato is complemented by its very valuable composition [4], explained by their very high antioxidant content. The most abundant antioxidant in tomato is lycopene (approx. 80% of all carotenoids) [5]. Thanks to its diverse techno-functional properties, a wide variety of products can be produced from tomato. Among the most demanded tomato-based products are the canned, concentrate, sauce, and ketchup [6], but tomato paste is the most commonly consumed.

The minimum quality requirements and ingredients that can be used in tomato processing are defined in local and international legislations and standards to protect consumer health and ensure fair food trade [7]. According to the Codex Alimentarius, “processed tomato concentrate is the product: prepared by concentrating the juice or pulp obtained from substantially sound, mature red tomatoes (*Lycopersicon*/*Lycopersicum esculentum*) strained or otherwise prepared to exclude the majority of skins, seeds and other coarse or hard substances in the finished product; and preserved by physical means”. In addition to tomato fruit, the ingredients permitted during processing are salt, spices, aromatic herbs, their natural extracts, lemon juice, and water [8]. Within the European Union, the additives that may be included in concentrates are strictly regulated. These include the maximum amount of acidity regulators and salt that the end-product can contain. In addition to the prohibition of colorants [9], all ingredients used in the manufacture of the product must be indicated on the label of the product package [10]. Despite these regulations, issues of food fraud continue to be on the rise typically, to reduce production costs or to produce products more appealing to consumers for increased profit. The dangers of economically motivated food adulteration (EMA) might be of higher risk than “traditional” food safety hazards because the contaminants are often unknown and unconventional [11]. In the case of fresh tomato, a common problem is the mislabeling of provenance or organic status. Addition of extra water or cheaper bulking agents (e.g., processing by-products, starch) can also occur during tomato juice and puree production. Other possible adulterants include sugars, acidity regulators, and even toxic synthetic dyes [12].

For the objective qualification of tomato products, soluble solid content (SSC) and Bostwick consistency determination are standard methods generally applied in the industry [13]. In the latter, the flow of a specified quantity of sample is measured [14,15] on the basis of which the consistency and shear-thinning behavior of the product can be inferred [16]. These are easy-to-apply methods but due to their low accuracy and lack of selectivity for the different adulterants, it is expedient to employ easy-to-use, rapid and high sensitivity analytical methods, in the control of different quality properties or food authentication [17,18]. Spectroscopic and multisensorial techniques, like near infrared spectroscopy (NIRS) and electronic tongue (e-tongue) have the potential to fulfil these requirements as, the attribute(s) in question could be determined simultaneously after calibration.

NIR spectroscopy is routinely used for the analysis of food components, functional and sensory properties, manufacturing intermediate, and end-products using the light wavelength range between 750–2500 nm [19]. NIRS proved to be an efficient method in the detection and prediction of mold (*Botrytis cinerea* or *Alternaria tenuissima*) contamination in tomato purees, through the identification of important wavelengths [20]. Several studies have focused on profiling and estimating the composition (water, sugar, acid, protein, salt, carotenoid content) or the acid/brix ratio of tomato juices [21,22,23,24]. The method was simultaneously used to determine acidity, solids (total and soluble), lycopene, and β-carotene content in tomato concentrate products [25,26]. Using principal component analysis (PCA) and soft independent modeling of class analogy (SIMCA), NIRS proved to be very accurate when tomato fruits of different ripening stages (green, pink, red) were classified [27]. For qualitative and quantitative purposes, various chemometric tools such as discriminant analysis (DA), cluster analysis (CA), k-nearest neighbor (KNN), SIMCA, partial least squares regression (PLSR), multiple linear regression (MLR), support vectors regression (SVR), artificial neural networks (ANN) have been used for NIRS spectral interpretations [28].

The e-tongue is a device capable of measuring simple or complex, soluble, non-volatile compounds. It has been widely used in fields where sensory monitoring cannot be implemented for some reasons such as testing the authenticity of products [29,30]. Researchers have repeatedly examined the suitability of e-tongue for determining tomato varieties and ripening stages. According to Liu et al. [31] tomato planted and harvested in- and off-season could be clearly distinguished on the basis of their taste profile with an e-tongue. With PCA, there was clear separation of the two sample groups along the second PC which, points to differences in the overall flavor. This is consistent with the results of sensory evaluation. For quantification of organic acids, individual sugars, and minerals in six Belgian tomato cultivars, a laboratory-made and a commercial e-tongue were compared for the differentiation of varieties based on taste profiles. The results showed that, the two e-tongues predicted the composition and sensory properties of the tested tomato samples with good accuracies [32]. Another study showed e-tongue combined with LDA was not only able to detect differences in sugar-acid (total soluble solids-titratable acidity: TSS/TA) ratio but also, could distinguish between commercial varieties harvested at six different maturity periods [33]. In a subsequent study, the authors examined the effect of blanching and refrigeration on the flavor profile of tomato. The fruits harvested in “light red” state showed a clear treatment-based separation. The refrigeration treatment induced more changes in the samples [34]. As correlative techniques, e-tongue and Attenuated total reflection- Fourier-transform infrared (ATR-FTIR) spectroscopy were applied to determine the main sugar and acid profile, measured by High Performance Liquid Chromatography (HPLC), of four tomato cultivars. The classification by canonical discriminant analysis (CDA) revealed that the cultivars were well grouped and this was confirmed by the independent validation, when discrimination was pursuant to organic acid content. The canonical correlation analysis (CCA) used to correlate the HPLC results with e-tongue and ATR-FTIR spectroscopy data showed correlation of 0.90 and 0.98 to the two different advanced techniques respectively [35].

The analytical rapid methods, applied in most of the researches summarized above, have been primarily used to map differences between varieties, fruit maturities, or to estimate unique quality traits (e.g., sugar, organic acid, lycopene content, consistency) of tomato products. Little or no scientific source is available on the authenticity of tomato concentrates, especially not for the comparison of conventional (i.e., SSC and Bostwick consistency tests) and e-tongue analysis, while for the NIR-based method, slightly more information is available. This study aimed to determine the efficiency of conventional (SSC and Bostwick consistency tests) and advanced methods (NIRS, e-tongue) in the detection and prediction of minimal concentrations of various common adulterants (ground paprika seed, corn starch, sucrose, salt) in tomato pastes.

## 2. Materials and Methods

### 2.1. Materials

Authentic tomato concentrate (paste) sampling was performed in a tomato processing plant and aseptically transported to the research laboratory for further analysis. The samples were from the same batch and belonged to the quality category of 28-30 degrees Brix (°Bx) in terms of water-soluble solid content. To mimic suspected tomato paste adulteration in the market, the samples, were mixed with ground paprika seed (P—Rubin Szegedi Paprikafeldolgozó Ltd., Szeged, Hungary), corn starch (K—Spar Magyarország Kereskedelmi Ltd., Bicske, Hungary), sucrose (C—1. MCM Ltd., Kaposvár, Hungary), and salt (S—Compex-Só Ltd., Nyirtass, Hungary). Five levels of adulteration (0.5%; 1%; 2%; 5%; 10%) were determined for paprika seed and starch, and four levels of adulteration (0.5%; 1%; 2%; 5%) for sucrose and salt. The total weight of each sample was 120 g. In this research, minimal adulteration was modelled and the concentration ranges were determined such that, they did not cause large-scale visible color change in the samples. On the other hand, they do not exceed concentration limits known from legislation [36]. Distilled water (W) was added to samples containing ground paprika seed (WP) or corn starch (WK) to avoid obvious consistency differences caused by the added adulterants, similarly, to perceived practices [37,38]. The powder-water ratio to be added was determined in preliminary experiments, which were performed with a rotational rheometer.

### 2.2. Methods

#### 2.2.1. Preliminary Experiments with Rotational Rheometer Development of Adulterant Mixtures

In preliminary experiments rotational rheometer, Haake RotoVisco (Thermo Scientific, Karsruhe, Germany) was applied to determine the viscosity of authentic and adulterated tomato paste samples. This way, we were able to set the powder-to-water ratio in the adulterated samples that was closest to the behavior of the authentic samples. With the rotation rheometer, the test substance was placed between two concentric cylinders. One cylinder was fixed and the other could be rotated with different angular velocities. Rotation of the cylinder tangentially deformed the material to be tested which, showed resistance to deformation. The first step was to homogenize the samples with the applied Z10 DIN Ti probe head with increasing shear rate up to 100 1/s during 60 s and with decreasing shear rate to 1 1/s during 60 s. After homogenization, the mixed samples were measured with increasing shear rate up to 100 1/s during 60 s; at the maximum shear rate during 100 s; finally shear rate decreased to 1 1/s during 60 s. The latent viscosity was determined in the constant shear rate stage.

**Figure 1:**
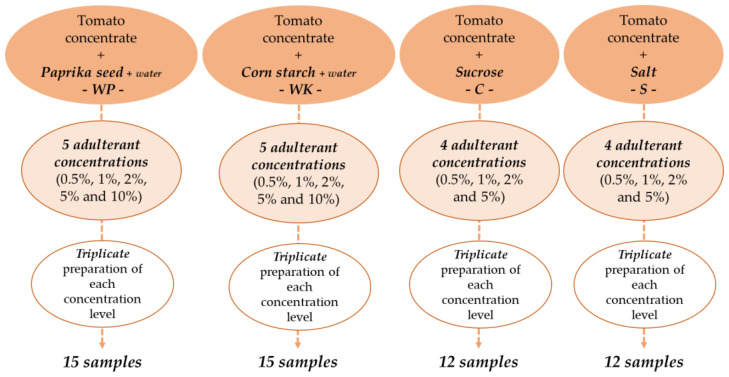
Preparation of adulterated tomato paste samples.

We chose the powder-water ratio at which the apparent viscosity of the adulterated tomato concentrate was closest to the authentic one. For the samples containing paprika seed, the weight of water added was 1.5 times the weight of the adulterant, for starch-containing samples, 0.5 times the weight of the adulterant. Accordingly, there was a total of 19 samples of different composition. Each sample was prepared in triplicates, resulting in a set of 57 samples, including the authentic ones (Figure 1). Until the measurements began, the samples were stored at 5 °C, then kept at constant room temperature during the measurements.

#### 2.2.2. Determination of Water-Soluble Solids Content

The water-soluble solid content (SSC) was determined with a digital pocket refractometer (PAL-1, ATAGO Co., Ltd., Tokyo, Japan). After calibration with distilled water, the instrument gave the dissolved solids content (SSC) in °Bx units based on the refractive index of the samples. The soluble solid content of both initial, authentic and adulterated tomato paste samples was measured and recorded. SSC gives soluble components in the samples, which primarily means sugar content in fruits and tomato products. Both SSC and sugar content are often reported in °Bx, so the two terms are interchangeable in this case [39].

#### 2.2.3. Determination of Bostwick Consistency

Based on the results of the soluble solid content, the authentic and adulterated tomato paste samples were diluted to 12.5% with distilled water for the consistency tests [40]. The samples were then loaded individually into the sample chamber of the Bostwick consistometer before releasing the gate of the equipment. After the release of the gate equipped with a spring on one side of the chamber, the sample spread (flowed) in one direction on a sloped surface due to its own weight. The distance covered by the samples was recorded after 30 s [37,38].

#### 2.2.4. Near Infrared Spectroscopy (NIRS) Measurements

The near infrared spectroscopy measurements of authentic and adulterated tomato paste samples were performed with a benchtop MetriNIR spectrometer (MetriNIR Research, Development and Service Co., Budapest, Hungary). The spectral data were collected in the wavelength range of 740–1700 nm. A thermoregulated cuvette with a sample layer thickness of 0.4 mm was used to maintain the temperature of the samples at 25 °C. The samples were measured in a randomized order and a sum of nine transflectance spectra were recorded for each adulteration level (three consecutive scans for all the three repeats).

#### 2.2.5. Electronic Tongue Measurements

The electronic tongue measurements of authentic and adulterated tomato concentrate samples were performed with an αAstree potentiometric electronic tongue (Alpha M.O.S., Toulouse, France) [41]. The ion-selective field-effect transistor (ISFET) sensor array developed for food testing (ZZ, JE, BB, CA, GA, HA and JB) versus the Ag/AgCl reference electrode were used to analyze the samples. In order to ensure the lucidity of the analysis, the electronic tongue was conditioned and calibrated according to the manufacturer’s recommendation before commencing the actual sample measurements. The instrument was equipped with a 16-position sample holder [41]. The authentic and adulterated tomato paste samples were one hundred-fold diluted before filtering with pleated paper filters with a pore size of 30 μm and diameter of 125 mm (Macherey-Nagel GmbH. & Co., Düren, Germany). For one hundred-fold dilution, 1.00 g of a sample was weighed into 100 cm^3^ volumetric flasks and filled up to mark with distilled water. During the measurements, the tested sample volume was 100 cm^3^, the sampling time was 120 s, the sampling frequency was 1 s, and the cleaning time with distilled water was 20 s. For these measurements, the initial sample size was reduced from 57 to 39, in order to moderate the memory effect and drift in e-tongue sensors. For this purpose, only samples with the minimum, middle and maximum adulterant concentrations were measured with the electronic tongue (Table 1). The selection of the samples to be tested was determined in such a way that, well-balanced experiments were maintained despite the limited number of positions and the samples that could be tested at the same time. The measurements were performed during three days. Each sample was prepared in three repeats and each repeat was measured 4 times, resulting in 12 data points for each sample.

#### 2.2.6. Statistical Analysis

The result of the SSC and the Bostwick consistency measurements of authentic and adulterated tomato concentrate samples were evaluated with univariate data analysis. First, descriptive statistics (mean, standard deviation) was used for primary characterization of the tested samples, then two-way analysis of variance (ANOVA), followed by pair-wised Tukey-HSD comparison at *p* < 0.05 significance level. Two-way ANOVA was used to detect if the adulterant types, adulterant concentrations and their interactions (adulterant × adulteration level) have significant effects on the SSC and Bostwick consistency of the samples. Significant differences within each adulterant group (paprika seed, corn starch, sucrose, salt) were indicated by different letters [42]. Those results which were not significantly different from each other have common letter(s), while those bearing different letters show significant difference [43].

Multivariate data analysis was employed on the results of NIRS and e-tongue. For the NIRS evaluation, the wavelength range of 950–1650 nm was taken into account, in order to reduce spectral noise. Savitzky-Golay smoothing (SG) filter with a second-order polynomial and 21 points was used to reduce the noise of the NIR spectra [44], and multiplicative scatter correction (MSC) was used to reduce baseline variation [45]. Exploratory qualitative evaluation was performed with principal component analysis (PCA), which allowed outlier detection, pattern identification and visualization. Linear discriminant analysis (LDA) was applied to develop classification models. LDA models were optimized based on principal component scores. First LDA model was developed for the classification of the authentic samples and the different types of adulterants regardless of their concentrations. Here the adulterants were used as categorical dependent variables (class variables) using the whole dataset. LDA models were then developed were destined to be differentiated between different concentration levels first using the whole dataset, then using the specific data sets corresponding to the adulterants. In this case the class variable was the concentration. The predictive accuracy of the LDA models were tested by dividing the data into a training (calibration) and test (validation) set. The training set contained two-third of the data consisting of the first and second replicates, while the validation set (the last third), contained the third replicates. The classification models were validated with threefold cross validation by performing the data splitting and model building three times in a row; this ensured that each sample was listed at least once in calibration and validation. The average recognition accuracy for the calibration and the average prediction accuracy for the cross-validation were calculated from the obtained validation tables. Partial least squares regression (PLSR) was used to predict the adulterant concentrations of the samples. The first PLSR model was built on the whole data set to predict tomato paste content irrespective of the adulterant type. In the sequel models were also built separately to predict paprika, starch and salt in tomato pastes. For these evaluations as well, the different data sets were divided into training and validation sections. The predictive accuracy of the PLSR models was tested with leave-one-sample-out cross-validation (LOSOCV). In this case three consecutive scans of a sample replicate were left out for the calibration model and the testing was performed on the previously omitted three scans. The accuracy of the PLSR models was given by the coefficient of determination (R^2^) and the root mean square error (RMSE) of training (C) and validation (CV). The number of latent variables used in the evaluation was determined based on the RMSECV.

For the e-tongue data analyses, the results were evaluated based on the average value of the sensor signals measured in the last 10 s. In this case, the chemical sensors did not provide the same signal due to the shift of the sensor signals, therefore, drift correction (additive correction relative to reference samples) was performed on the raw data to mitigate the drift between the measuring days [46,47]. PCA was employed to detect and eliminate outliers. The subsequent LDA was performed as described for NIRS evaluations. Tomato paste sample composition predictions was also performed using the e-tongue data, in which leave-one-out cross-validation (“LOOCV”) was used. In this case, the model construction and validation were performed by omitting one event of the measurement data and testing with the remaining one case. This splitting had to be performed in a number fitting to the number of cases, thereby ensuring that all cases were included in the validation. Following to the date pretreatment detailed above mean-centering of the data was applied before the multivariate modelling. The data evaluation was done with Microsoft Excel, R-project (3.5.2) software and the “aquap2” package [48].

## 3. Results

### 3.1. Results of Conventional Methods

#### 3.1.1. Results of Soluble Solid Content Determination

The average soluble solid content of authentic tomato paste samples containing different adulterants are summarized in Table 2. The SSC of the authentic samples was 30.967 ± 1.358 °Bx. In general, addition of paprika seed or starch decreased the SSC, while addition of sucrose and salt increased it. Based on the two-way ANOVA test, the SSC of the authentic and the adulterated samples at various concentrations was significantly affected by the adulterant (*p* < 0.001), the adulteration level (*p* < 0.015), and their interaction (*p* < 0.01). Comparing the adulterant groups in pairs, showed strongly significant differences (*p* < 0.05) between the WP-C, WP-S, WK-C and WK-S adulterated samples. Examining the adulterations separately, it was found that the 10% paprika seed adulterated samples had a significantly different SSC compared to the samples adulterated at 0.5 and 1% adulteration levels. This was also typical in the case of samples containing starch, where 5–10% adulteration were significantly different. In the case of samples sucrose-falsified, there was significant difference between the 1% and 5% adulterated samples. However, there was no detectable difference in the samples containing salt.

#### 3.1.2. Results of Bostwick Consistency Determination

The average Bostwick consistencies of authentic and adulterated tomato pastes are summarized in Table 3. The consistency of the authentic samples was around 5.833 ± 0.577 cm/30 s. Based on the results of the tests performed on the samples containing only ground paprika seeds, the extent of spreading slightly decreased with the gradual increase in the adulteration levels. This trend was also observed for samples adulterated with corn starch. In contrast, the flowing distance over time clearly increased as a result of sucrose adulteration. Salt addition slightly increased the flow rate of the samples. The two-way ANOVA test performed on the entire dataset revealed that the adulterant (*p* < 0.001), the adulteration level (*p* < 0.01), and their interaction (*p* < 0.01) had an equally significant influence on sample consistency. By the pair-wised comparison of adulterants, the samples adulterated with glucose, showed significant difference (*p* < 0.01), but salt adulterated samples did not. Samples adulterated with salt differed significantly only from the starch-containing ones. Analyzing the adulterations respectively, it was ascertained that the gradual paprika seed, starch or salt addition did not induce demonstrable consistency change(s) in tomato pastes. In the case of sucrose, the maximal 5% adulteration resulted in significantly distinguishing alteration in textural properties compared to all other adulteration levels.

### 3.2. Results of Rapid Analytical Methods

#### 3.2.1. Results of Near-Infrared Spectroscopic Analysis

##### PCA and LDA Results of NIRS Analysis

**Figure 2:**
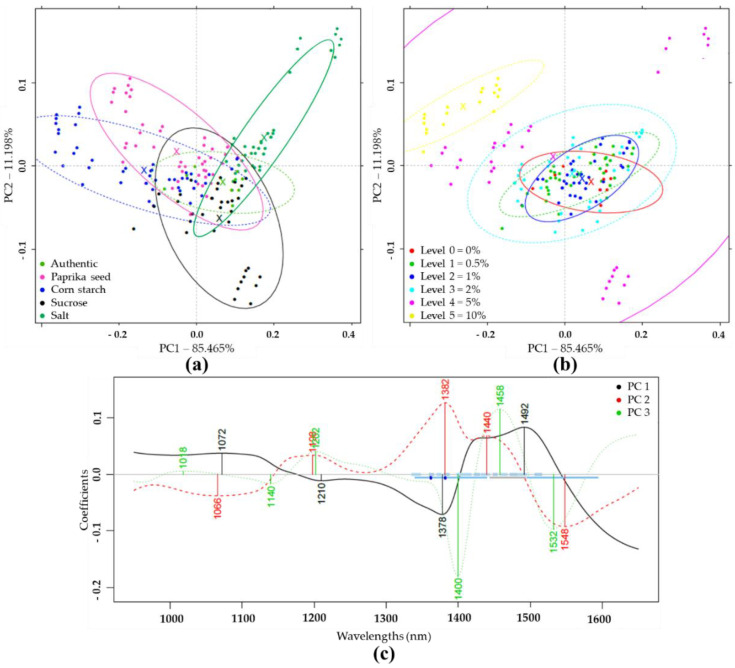
Principal component analysis (PCA) on the whole Near Infrared Spectroscopy (NIRS) dataset (N = 171) in the wavelength range of 950–1650 nm, after Savitzky-Golay smoothing (SG) and multiplicative scatter correction (MSC) pretreatment of the NIRS spectra: (**a**) Score plot by adulterant type; (**b**) Score plot by adulteration level; (**c**) PCA loadings.

Based on PCA analysis of pretreated NIR spectra of authentic and differently adulterated samples, the first two principal components (PC1, PC2) described 96.66% of the total variance (Figure 2a). Within the adulterant groups, the increasing concentrations gradually shifted from the center to the extremities of the plot. The higher concentration levels were visually more separated than the lower concentrations (Figure 2b). Figure 2c demonstrates the wavelengths that contributed most to the formation of the first three principal components. In this plot, the absorption bands important for discrimination could be identified.

**Figure 3:**
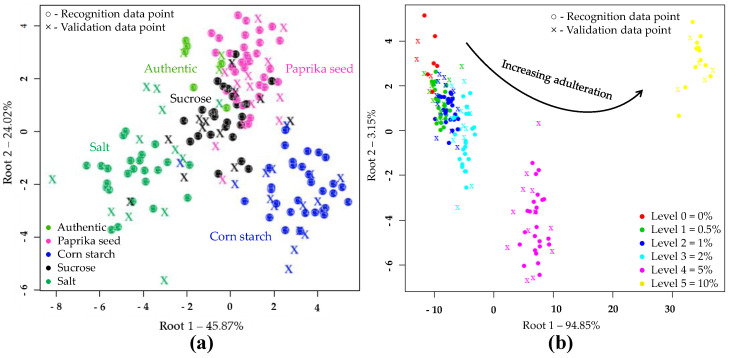
Linear discriminant analysis (LDA) classifications on the whole NIRS dataset (N = 171) in the wavelength range of 950-1650 nm, after SG, MSC pretreatment of the NIRS spectra and three-fold cross-validation: (**a**) LDA plot when the adulterant type was used as class variable (C0–C5 present the adulterant concentration); (**b**) LDA plot when the adulterant concentration was used as class variable.

The results of LDA models built to classify samples based on the type of adulteration are shown in Figure 3a. During the LDA modeling, 50 PC’s were used. According to this, the different adulterants were clearly separated. The sharpest grouping was observed for the authentic and salt-adulterated samples. During the model construction, the data points showed no overlapping, and resulted in average training and validation accuracies of 100% and 86.68%, respectively when, the adulterant type was used as class variable in the whole dataset. In cross-validation, the data points corresponding to authentic tomato pastes were classified with an accuracy of 100%. There was also a very good classification accuracy for salt (91.67%) and starch-containing samples (88.87%). In the case of paprika seed and sucrose 77.8% and 75.06% correct classifications were obtained during validation, respectively. Table 4 summarizes where misclassification might have occurred presumably due to the low adulterant concentrations.

Figure 3b confirms the accuracy with which increasing levels of adulteration could be classified, regardless of the adulterant involved. The separation of concentration levels can be observed almost on a curve. The average correct recognition and prediction accuracies were 97.92% and 83.79%. The LDA model distinguished authentic, 5 and 10% adulterated samples with classification 100% accuracy during both model building and validation. Adulteration levels of 0.5, 1 and 2% were classified with accuracies of over 93% during model building and over 55% during validation.

The accuracy with which the different adulterant concentrations could be classified was determined separately in the data sets filtered on the basis of adulterants. The detailed LDA classification results are displayed in Table 5 and Table 6.

From Table 5, the classification models of authentic samples and samples adulterated at 10% were generally the most accurate. For each adulterant, different concentration levels were identified with 100% accuracy during model construction. For samples adulterated with paprika seed, the average correct classification was 86.83% after three-fold cross-validation. There was no misclassification in the samples containing 5 or 10% ground paprika seed. In the case of 2% adulteration, 11% of the samples were misclassified as belonging to 1% adulteration group, while at 1% adulteration, 11.04% were misclassified as lower (0.5%) and upper (2%) concentrations. The model correctly classified 55.67% of the data points representing the minimum adulterant concentration (0.5%). For starch-adulteration, the classification accuracy was 78.64% for validation. 100% correct classification was obtained only at 10% adulteration. Samples containing 2% and 5% adulterant, were classified with an accuracy of 89%, and 11% were misclassified as belonging to the lower concentration levels. In the case of 1% adulteration, the average correct classification was 66.89%. During model validation, the lowest adulterant concentration (0.5%) was 33.33% correctly classified. These samples were typically misclassified as authentic in 55.67% of the cases.

From Table 6, the validation accuracy of sucrose-adulterated samples was 76.94%. There was no misclassification in the samples containing 5% sucrose. Accurate classification was obtained in 2% adulteration (89%). Misclassifications of 33.33% and 11% of the data points corresponding to 2% and 0.5% concentrations was observed in the model built to classify 1% adulteration. The minimum concentration (0.5%) was determined with an accuracy of 44.48%. The other part was misclassified as authentic (44.48%) and adulterated in 1%. The most precise classification was achieved in the salt-containing samples. The average correct classification was 97.65%. There was little misclassification between authentic and minimally falsified samples. The latter was predicted with an accuracy of 89%.

##### PLSR Results of NIRS Analysis

The predictive PLSR models built to predict the adulterant contents in the tomato paste mixtures are shown in Table 7. The coefficients of determination (R^2^) and root mean square errors (RMSE) describing the model fit were higher than 0.96 and lower than 1% *w*/*w*, respectively, during both training and validation. The method proved to be particularly sensitive to paprika seed and salt contamination. In the course of validation, the values of R^2^_CV_ were higher than 0.99 and the RMSECV were smaller than 0.5%. The global model built to determine tomato paste content showed the least accurate prediction (RMSECV = 0.886).

Based on the PLS regression vectors, the wavelengths that mostly contributed to the effective model constructions were identified. These major wavelengths, included in Table 8, are associated with the special vibrational bands of various functional groups. The C-H, N-H and O-H bands are characteristic of the first and second overtone region. Besides, the NIR spectra could be altered by the so-called fingerprint frequencies, which could arise from entire molecular vibrations [49]. Seeing that several types of adulterants might have caused the same decrease in tomato paste content in the examined samples, in this case manifold spectral changes could be traced during estimation, especially in the first overtone of O-H stretching vibrations.

#### 3.2.2. Results of Electronic Tongue Analysis

##### PCA and LDA Results of E-Tongue Analysis

**Figure 4:**
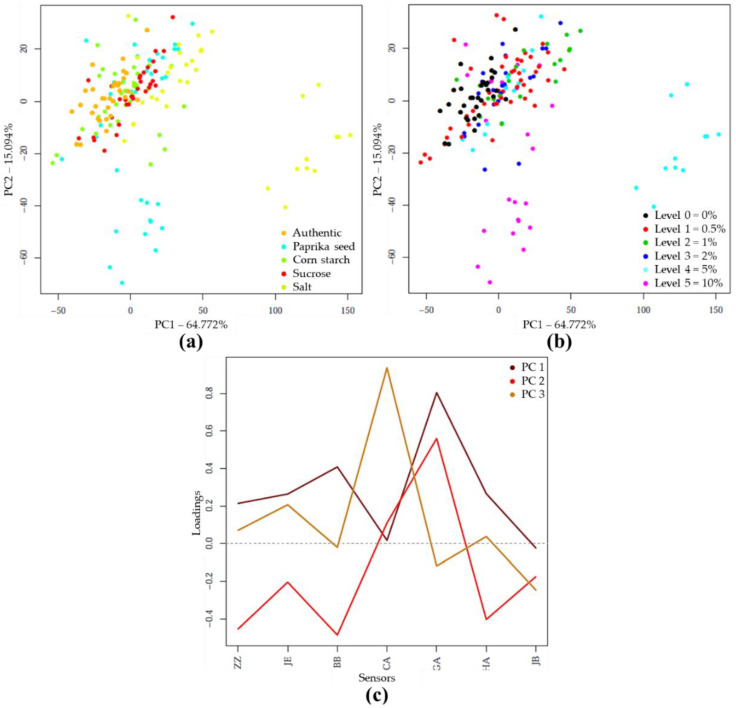
PCA on the whole e-tongue dataset (N = 262) after drift correction and outlier detection: (**a**) Score plot by adulterant type; (**b**) Score plot by adulteration level; (**c**) PCA loadings.

The PCA analysis on the e-tongue sensor signals of authentic and adulterated tomato paste samples showed that the first two principal components (PC) described nearly 80% of the total variance. The separation of different types of adulteration was observed along both PC1 and PC2, due to the varying concentration levels (Figure 4a). Visually, the higher concentrations were more separated (Figure 4b). Figure 4c illustrates, for the first 3 principal components, the extent to which the seven sensors contributed to distinguishing between authentic and adulterated samples. Consequently, the prominent sensors could be identified

**Figure 5:**
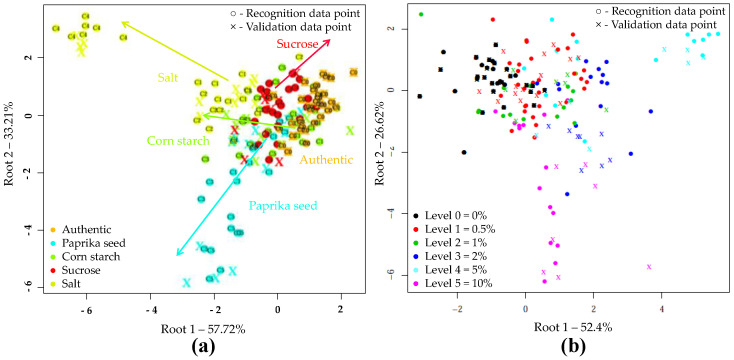
LDA classification model on the whole e-tongue dataset (N = 262) after drift correction, outlier detection and triple cross-validation: (**a**) LDA plot when the adulterant type was used as class variable (C0–C5 present the adulterant concentration); (**b**) LDA plot when the adulterant concentration was used as class variable.

The supervised detection and classification of the different adulterants during LDA recognition and validation are illustrated in Figure 5a. In this LDA evaluation, five principal components were employed. For e-tongue the data points showed significant overlap. The average correct classification was 60.73% and 52.56% in model construction and testing (Table 9). The data points representing the authentic samples were classified most accurately (98.04%, 97.66%). A small percentage was misclassified belonging to the starch-containing samples. The classification accuracies of samples adulterated with paprika seed or salt were 61.78% for model building and 58.8% for testing. In the case of starch adulteration, these values were 61.76 and 41.18%, for calibration and validation. For the latter, 41.18% were misclassified as unadulterated. In the case of sucrose adulteration, only 20.3 and 6.28% of the samples were classified correctly, the majority (67.18%, 78.07%) was clustered to the authentic set. Figure 5b illustrates the classification according to the concentration levels, regardless of the adulterant type. The average correct classification during training and validation were 63.68% and 53.79%, respectively. The method most accurately distinguished the authentic, 2 and 10% adulterated samples. In these cases, the classification accuracies were between 63.64% and 93.75% for recognition, and 63.62% and 87.51% for prediction.

In the case of the e-tongue, we also examined in more depth how accurately the different concentration levels could be classified with LDA. In these LDA evaluations, three principal components were employed. These results are integrated in Table 10 and Table 11. The data points corresponding to the different adulterant concentrations showed significant overlap during model construction and validation. In general, the applied methods effectively identified the authentic samples. The average correct classifications of the samples falsified with paprika seed were 90.59% and 75.72% for prediction and validation. There was no misclassification in the samples containing 10% adulterant. For 2% adulteration, the classification accuracies were 86.36 and 45.5%. In the latter case, most of the misclassification occurred at the lower concentrations. The minimal adulteration (0.5%) was detected with an accuracy of 79.12% and 66.75%. During validation, misclassification was observed in the authentic (25%) and 2% adulterated samples (8.25%). For starch-falsified samples, the average correct classifications were 84.89 and 54.28%, during training and testing. The 10% adulteration was classified with an accuracy of 90.87 and 18.26%. In the latter case, there was a significant misclassification to the authentic sample cluster (45.5%). The 2% adulteration was correctly classified in 95.88 and 75%. Misclassification was mostly for the 10% adulteration. At minimal concertation (0.5%) detection, 59.07 and 36.34% correctness were obtained, the LDA classified a large proportion of the samples as authentic.

As Table 11 summarizes, sucrose adulteration was predicted with 80.22% and 56.39% accuracies. The maximum adulteration (5%) was classified correctly with accuracies of 81.86% and 45.5%, during model building and testing, respectively. In the latter case, 45.5% of the samples were classified as adulterated in 0.5%. At 1% adulteration, 77.25% and 36.24% of the corresponding samples were well identified in model training and validation. The misclassifications were dominantly in the lower concentration levels. The average correct classification of minimal adulteration (0.5%) was 64.92% for model building and 50% for validation. A total of 40.12% was misclassified for higher adulteration levels. In the case of salt-containing samples, 94.49% and 86.16% correct classifications were achieved. There was no misclassification at the maximum salt content (5%). The 1% adulteration was classified with an accuracy of 86.36% and 72.75%, in model building and validation. The data points corresponding to the minimally adulterated samples (0.5%) were detected correctly in 91.62 and 75%, when misclassification occurred for the samples falsified in 1%.

##### PLSR Results of E-Tongue Analysis

PLSR was also performed on the e-tongue data to determine the accuracy with which the degree of adulteration could be predicted. Table 12 summarizes the main coefficients of the evaluation which ascribes the fit of the built models. The least accurate fit was found in sucrose and starch concentration prediction. The coefficients of determination and root mean square errors were between 0.33–0.51%*w*/*w* and 1.38–2.57%*w*/*w*, respectively during validation. The most accurate fit was achieved in the cases of salt and paprika seed adulteration. The coefficients of determination were higher than 0.93 and the RMSE less than 1%*w*/*w*, especially for the salt-containing samples. The PLSR model for tomato paste content prediction, constructed on the whole dataset, resulted in an R^2^ of 0.79 and 0.77 for prediction and validation. The tomato paste content was predicted with the highest error in both calibration (RMSEC = 2.63%*w*/*w*) and after cross validation (RMSECV = 2.7%*w*/*w*).

## 4. Discussion

### 4.1. Results of Conventional Methods

#### 4.1.1. Soluble Solid Content

In tomato concentrate categorization, soluble solid content is a generally accepted quality parameter in the industrial practice. The SSC value of the tested authentic samples was around 30.967, which corresponds to a minimum of 24% natural total soluble solids, so the name “tomato paste” can be used [8]. However, this value is more typical for concentrates produced by the hot break process [50,51]. In the hot break process, the cleaned tomatoes are crushed at a temperature between 90–100 °C, extracted, refined and the resulting mass is concentrated. The paprika seed (industrial by-product) and starch dosage supported the expectation that slight SSC dilution can be caused by them when added to pastes. Based on the ANOVA results, significant differences were detected in samples containing a higher proportion of adulterants (5 and 10% adulteration). Under the influence of sucrose and salt, the maximum adulteration showed a considerable increase in SSC, as confirmed in literature [12,52], however there was a demonstrable significant difference in the case of 5% sucrose content.

#### 4.1.2. Bostwick Consistency

Consistency is the second most important quality attribute after color in consumer perception, so it is often measured at different points during tomato production and storage. The average consistency of the authentic samples was around 5.833 cm/30 s, which falls in the consistency range, typical of hot break products. At the temperature used for hot break, the pectolytic enzymes are inactivated, consequently, the pH and viscosity of tomato serum are higher [53]. The consistency of tomato pastes produced with cold break process is generally 8.0–11.0 cm/30 s [50]. Due to paprika seed and starch adulteration, a slight thickening was seen in the consistency results, but no significant difference was detected compared to the authentic samples. This is due to the fact that water was also added to these samples during sample preparation. McCarthy, Sacher, and Garvey (2008) tracked the flow properties of tomato paste blends and ketchups made from them, which was associated with Bostwick consistency. Consistency ranged from 1.3 to 7.2 cm/30 s for the pastes [38]. This has been supported and confirmed in literature. For example, in the presence of starch [54] and hydrocolloids [55], the spreading time of tomato sauces is reduced. This can be attributed to the interaction with water, which leads to the formation of a gel-like structure [56]. In comparison, the increase in sucrose concentration resulted in an abrupt spreading in our research. This was most pronounced at 5% adulteration. In the case of salt adulteration, an increase in the spreading time was also observed, but it was not statistically significant. The effect of excess electrolytes from salt (NaCl) solution, on the viscosity of tomato juices has also been reported in literature. It was found that NaCl decreases the charge on the pectin molecules, hence, the formation of dimers and trimers resulting in growth of the pectin molecule folds. In addition, the number of hydrogen bonds decreases and consequently the consistency decreases [13].

The aforementioned standard methods together were generally incapable of detecting adulteration in the demonstrated measurement arrangement. If there was a significant difference, it was most pronounced in the case of higher adulterant concentration (5–10% adulteration). Since these means are capable of yielding a simple result, the application of fingerprint-like methods was well-founded. This way we could see more deeply the patterns hidden in the tomato paste blends.

### 4.2. Results of Analytical Rapid Methods

#### 4.2.1. Near-Infrared Spectroscopic Analysis

##### PCA and LDA Results with NIRS

NIR spectroscopy proved to be very effective in detecting, identifying and predicting adulterants in our measurement arrangement. During calibrations, LDA correctly classified adulterants with 100% accuracy while the validation accuracy was 86.68% (Table 4). The authentic samples were classified with 100% accuracy. Examining the effect of adulterant concentrations separately showed similarly good results. In the course of model building, the mean correct classification was 100% in all cases, when the adulterant concentration was used as the class variable. During validation, 86.83, 78.64, 76.94 and 97.65% average classification accuracies were achieved for paprika seed, starch, sucrose and salt adulteration. In a 2009 study, researchers determined the accuracy of changes in tomato juices over a one-month period using NIR transmittance spectroscopy and various classification methods. They obtained 100% accuracy with LS-SVM, 97% with DPLS (discriminant partial least-squares), 90% with SIMCA, and 95.5% with DA [57].

##### PLSR results with NIRS

Based on the pretreated spectra and PLSR, the concentration of adulterants in the tomato pastes could be predicted with high accuracy (R^2^ > 0.96) and minimal error (RMSE < 1%*w*/*w*), during both model building and validation. Similarly, good results were obtained when the feasibility of multispectral imaging (405–970 nm) was evaluated by comparing the results of PLSR, LS-SVM, and BPNN to detect and predict minimal sucrose (analytical grade or sugarcane) adulteration (1–9%*w*/*w*) in tomato pastes. The best prediction was achieved by the LS-SVM model with R^2^ of 0.936 and 0.966, and RMSEP of 0.521% and 0.445% for the two batches (30 and 36 °Bx), respectively. The applied methods classified minimal sugar-adulterated samples (1%) with 100% accuracy. The wavelengths that contributed most to the estimation were as follows for the samples adulterated with analytical grade sucrose: 630, 645, 700, 780 and 850 nm [58]. A previous study on mold (*Botrytis cinerea*, *Alternaria tenuissima*) detection and prediction in tomato purees also identified the wavelengths associated with the absorption bands of chitin which is the major cell wall component of molds [20]. The wavelengths prominent for our estimation were all identified (Table 8). Wavelengths that can be associated with N-H stretch 2nd overtone, -CH_3_, >CH_2_ 2nd overtone, C-H combination bands 1st overtone, O-H and N-H 1st overtone. These can be explained by the presence of proteins, carbohydrates, and lipids in the seeds. The following vibration modes dominated the starch estimation: C-H 2nd overtone, C-H combination bands 1st overtone, O-H and C-H 1st overtone. The absorption of carbohydrates can be attributed to these wavelengths. In the case of sucrose adulteration, the important spectral regions nearly coincided with the starch. As a result of salt addition alterations were observed in the 2nd overtone region of O-H, N-H and C-H, 1st overtone of C-H combination, 1st overtone of O-H and N-H [49,59,60]. There was great variability in the spectra in the 1st overtone region of water (1300–1600 nm). The studying of this range falls under the aquaphotomics approach [61].

#### 4.2.2. E-Tongue Analysis

##### PCA and LDA Results with E-Tongue

The e-tongue analysis was characterized by reduced efficiency in identifying and predicting various adulterants in tomato pastes. There was significant overlap in the data points mainly at the lower adulterant concentration levels. In the course of recognition and validation, LDA classified the different adulterants with 60.73 and 52.56% accuracies (Table 9). The authentic samples were distinguished with 98.04 and 97.66% in model building and testing. When the adulteration level classification was run on the same data set, the discrimination was not explicitly consistent. This can be attributed to the fact that a reduced number of samples were analyzed with the e-tongue, accordingly not all concentration levels were present. Examining the effects of adulterants on the sensor signals, it can be said that the LDA was the most accurate to classify the concentrations of samples containing salt and ground paprika seed. The average correct classifications were the following for recognition and validation in the former case: 94.49 and 86.16%; and in the latter case 90.59 and 75.72%. Beullens et al. [32] used a self-developed and a commercially available e-tongue to classify six Belgian tomato cultivars based on their taste profiles. The CDA was able to sharply distinguish between different varieties. Hong and Wang [62] also used e-tongue coupled with PCA and CDA to monitor the freshness of cherry tomatoes. Although the sample groups were well separated, no clear trend could be drawn regarding the storage time. This might be due to the complex metabolic processes that take place in the tomato fruits. The aforementioned authors classified different levels of adulteration in tomato juice. Fresh tomato juices were adulterated with overripe tomato juices in 10, 20 and 30%*w*/*w*. The method completely differentiated each concentration level [63].

##### PLSR Results with E-Tongue

The analogy summarized in the previous section considering classification can also be observed in PLSR models based on the e-tongue data, according to which the technique performs particularly poorly in estimating the composition of carbohydrate-containing tomato paste mixtures. The predictive models calculated paprika seed and salt content with high accuracies. In these cases, the coefficients of determination were higher than 0.93 and root mean square errors lower than 1%*w*/*w*. This phenomenon can be explained by the fact that e-tongue is basically suitable for measuring electrolyte liquids [64]. The researches mentioned in the previous subsection also determined how accurately certain quality traits could be estimated with e-tongue. The e-tongue developed by Beullens et al. [32] estimated simple sugars, organic acids and metal ions with minimal error and a correlation of nearly 1. In tomato freshness monitoring, the pH, SSC and vitamin C were predicted with root mean square errors of 0.099, 0.659 and 1.546 mg/100 g, respectively [62]. In another study, when the effect of overripe tomato juice adulteration on cherry tomato juice was evaluated, the pH and SSC were also measured and estimated. PCR predicted the pH with R^2^ of 0.9971 and RMSE of 0.0062, and predicted the SSC with R^2^ of 0.9946 and RMSE of 0.1419 [63].

## 5. Conclusions

The results highlight that the aforementioned conventional methods (SSC and Bostwick consistency tests) alone are not suitable for detection. If the SSC or Bostwick-consistency tests demonstrated significant difference, it was representative in the case of higher adulterant content (5–10% adulteration). NIR spectroscopy and e-tongue were proven to be much more sensitive to differentiate between different adulterants (ground paprika seed, corn starch, sucrose, salt) and concentrations. For the samples we examined, the NIR approach gave more accurate and promising results compared to e-tongue. Its detection and prediction were characterized by high accuracy. The applied non-destructive techniques and related chemometric analysis provide objective results rapidly and effectively for revealing adulteration. Despite the fact that the standard methods did not significantly contribute to accurate detection of food adulteration, their results can be used as a reference for the development of additional NIRS and e-tongue models based on the multivariate data to predict the parameters used in tomato paste grading practices.

## Figures and Tables

**Table 1:** Electronic tongue measurement samples.

Adulterants	Analyzed Concentrations (%*w*/*w*)					
Paprika seed	0	0.5	-	2	-	10
Corn starch	0	0.5	-	2	-	10
Sucrose	0	0.5	1	-	5	-
Salt	0	0.5	1	-	5	-

**Table 2:** Average soluble solid content and standard deviation of tomato pastes by adulterants and concentration levels—in °Bx.

Adulterant Concentration		Paprika Seed	Corn Starch	Sucrose	Salt
		-WP-	-WK-	-C-	-S-
0.	0%	30.967 ± 1.358 ^a, b^	30.967 ± 1.358 ^a, b^	30.967 ± 1.358 ^a^	30.967 ± 1.358 ^a^
1.	0.5%	31.200 ± 1.015 ^a^	31.833 ± 0.153 ^a^	32.167 ± 0.611 ^a, b^	31.733 ± 0.493 ^a^
2.	1%	31.900 ± 0.557 ^a^	31.433 ± 0.551 ^a^	31.500 ± 1.664 ^a^	33.367 ± 1.419 ^a^
3.	2%	30.100 ± 1.153 ^a, b^	30.233 ± 0.404 ^a, b^	32.067 ± 1.966 ^a, b^	30.033 ± 4.629 ^a^
4.	5%	30.133 ± 0.493 ^a, b^	28.900 ± 0.436 ^b^	35.967 ± 2.136 ^b^	34.467 ± 2.203 ^a^
5.	10%	28.367 ± 1.193 ^b^	28.933 ± 0.896 ^b^	—	—

^a, b^—letters assigning significant differences within adulterant groups among the level of adulterant concentration (columns).

**Table 3:** Average consistency of tomato pastes by adulterants and concentration levels—in cm/30 s.

Adulterant Concentration		Paprika Seed	Corn Starch	Sucrose	Salt
		-WP-	-WK-	-C-	-S-
0.	0%	5.833 ± 0.577 ^a^	5.833 ± 0.577 ^a^	5.833 ± 0.577 ^a^	5.833 ± 0.577 ^a^
1.	0.5%	6.533 ± 0.950 ^a^	7.00 ± 0.781 ^a^	6.733 ± 0.929 ^a^	7.500 ± 0.500 ^a^
2.	1%	7.033 ± 0.473 ^a^	6.633 ± 1.415 ^a^	7.133 ± 0.635 ^a^	7.467 ± 0.896 ^a^
3.	2%	5.933 ± 0.757 ^a^	5.933 ± 0.058 ^a^	7.167 ± 0.503 ^a^	6.100 ± 1.389 ^a^
4.	5%	6.467 ± 0.252 ^a^	6.000 ± 0.500 ^a^	10.433 ± 1.582 ^b^	8.100 ± 0.755 ^a^
5.	10%	6.300 ± 0.265 ^a^	5.567 ± 0.751 ^a^	—	—

^a, b^—letters assigning significant differences within adulterant groups (columns).

**Table 4:** Linear discriminant analysis (LDA) classifications on the whole Near Infrared Spectroscopy (NIRS) dataset in the wavelength range of 950–1650 nm, after Savitzky-Golay smoothing (SG) and multiplicative scatter correction (MSC) pretreatments and three-fold cross-validation when the adulterant type was used as class variable.

Accuracy		Authentic	Paprika Seed	Corn Starch	Sucrose	Salt	Average Classification
Recognition	Authentic	**100**	0	0	0	0	100%
	Paprika seed	0	**100**	0	0	0	
	Corn starch	0	0	**100**	0	0	
	Sucrose	0	0	0	**100**	0	
	Salt	0	0	0	0	**100**	
Validation	Authentic	**100**	6.67	0	2.75	0	86.68%
	Paprika seed	0	**77.8**	4.47	11.09	5.58	
	Corn starch	0	6.67	**88.87**	2.75	0	
	Sucrose	0	6.67	4.47	**75.06**	2.75	
	Salt	0	2.2	2.2	8.34	**91.67**	

**Table 5:** LDA classification after three-fold cross-validation and SG and MSC pretreated NIRS spectra when the adulterant concentration was used as class variable on paprika seed and starch dataset.

Adulterant	Accuracy		0%	0.5%	1%	2%	5%	Average Classification
Paprika seed	Recognition	0%	**100**	0	0	0	0	100%
		0.5%	0	**100**	0	0	0	
		1%	0	0	**100**	0	0	
		2%	0	0	0	**100**	0	
		5%	0	0	0	0	**100**	
		10%	0	0	0	0	0	
	Validation	0%	**98.4**	11	0	0	0	86.83%
		0.5%	1.6	**55.67**	11.04	0	0	
		1%	0	33.33	**77.93**	11	0	
		2%	0	0	11.04	**89**	0	
		5%	0	0	0	0	**100**	
		10%	0	0	0	0	0	
Corn starch	Recognition	0%	**100**	0	0	0	0	100%
		0.5%	0	**100**	0	0	0	
		1%	0	0	**100**	0	0	
		2%	0	0	0	**100**	0	
		5%	0	0	0	0	**100**	
		10%	0	0	0	0	0	
	Validation	0%	**93.64**	55.67	11.04	0	0	78.64%
		0.5%	3.98	**33.33**	11.04	0	0	
		1%	2.38	0	**66.89**	11	0	
		2%	0	11	11.04	**89**	11	
		5%	0	0	0	0	**89**	
		10%	0	0	0	0	0	

**Table 6:** LDA classification after three-fold cross-validation and SG and MSC pretreated NIRS spectra when the adulterant concentration was used as class variable on sucrose and salt dataset.

Adulterant	Accuracy		0%	0.5%	1%	2%	5%	Average Classification
Sucrose	Recognition	0%	**100**	0	0	0	0	100%
		0.5%	0	**100**	0	0	0	
		1%	0	0	**100**	0	0	
		2%	0	0	0	**100**	0	
		5%	0	0	0	0	**100**	
	Validation	0%	**95.56**	44.48	0	0	0	76.94%
		0.5%	3.71	**44.48**	11	0	0	
		1%	0.73	11.04	**55.67**	11	0	
		2%	0	0	33.33	**89**	0	
		5%	0	0	0	0	**100**	
Salt	Recognition	0%	**100**	0	0	0	0	100%
		0.5%	0	**100**	0	0	0	
		1%	0	0	**100**	0	0	
		2%	0	0	0	**100**	0	
		5%	0	0	0	0	**100**	
	Validation	0%	**99.27**	11	0	0	0	97.65%
		0.5%	0.73	**89**	0	0	0	
		1%	0	0	**100**	0	0	
		2%	0	0	0	**100**	0	
		5%	0	0	0	0	**100**	

**Table 7:** Adulterant concentration prediction with PLSR and leave-one-sample-out cross-validation (LOSOCV) validation on pre-treated NIRS spectra of authentic and adulterated tomato pastes in the wavelength range of 950–1690 nm.

Constituent	R^2^_C_	RMSE_C_ (%*w*/*w*)	R^2^_CV_	RMSE_CV_ (%*w*/*w*)	LV	N
Paprika seed	0.9953	0.238	0.9849	0.429	6	54
Corn starch	0.9897	0.354	0.9679	0.626	6	54
Sucrose	0.9887	0.189	0.9668	0.324	5	45
Salt	0.9937	0.141	0.9835	0.228	5	45
Tomato	0.9906	0.602	0.9796	0.886	14	171

R^2^_C_, R^2^_CV_—Coefficient of determination of model building and validation; RMSEC, RMSECV—Root mean square error of calibration and validation; LV—Latent variables; N—Sample count.

**Table 8:** Relevant wavelengths in the adulterant concentration predictions with PLSR and “LOSOCV” validation on pre-treated NIRS spectra of authentic and adulterated tomato pastes.

Constituent	Wavelengths (nm)	
Paprika seed	-	988, 1042, 1114, 1156, 1210, 1272, 1374, 1404, 1430, 1454, 1504, 1584
Corn starch	-	1108, 1158, 1270, 1380, 1416, 1490, 1518, 1560, 1590, 1612
Sucrose	-	1020, 1160, 1324, 1378, 1418, 1484, 1532, 1586
Salt	-	994, 1136, 1188, 1306, 1366, 1398, 1428, 1480, 1532, 1588
Tomato paste	-	1016, 1068, 1138, 1172, 1208, 1246, 1316, 1344, 1370, 1408, 1430, 1446, 1462, 1480, 1500, 1518, 1532, 1550, 1568, 1600

**Table 9:** LDA classifications after e-tongue data drift correction, outlier detection and three-fold cross—validation when the adulterant type was used as class variable.

Accuracy		Authentic	Paprika Seed	Corn Starch	Sucrose	Salt	Average Classification
Recognition	Authentic	**98.04**	19.11	35.29	67.18	8.83	60.73%
	Paprika seed	0	**61.78**	0	0	0	
	Corn starch	1.96	14.7	**61.76**	12.52	5.87	
	Sucrose	0	4.41	2.96	**20.3**	23.52	
	Salt	0	0	0	0	**61.78**	
Validation	Authentic	**97.66**	17.64	41.18	78.07	5.91	52.56%
	Paprika seed	0	**58.82**	8.82	0	2.91	
	Corn starch	2.34	17.64	**41.18**	15.65	11.74	
	Sucrose	0	5.91	8.82	**6.28**	20.56	
	Salt	0	0	0	0	**58.87**	

**Table 10:** LDA classifications after drift correction, outlier detection and three-fold cross-validation when the adulterant concentration was used as class variable on the e-tongue data of paprika seed—and starch—adulterated tomato pastes.

Adulterant	Accuracy		0%	0.5%	2%	10%	Average Classification
Paprika seed	Recognition	0%	**96.86**	20.88	4.5	0	90.59%
		0.5%	3.14	**79.12**	9.14	0	
		2%	0	0	**86.36**	0	
		10%	0	0	0	**100**	
	Validation	0%	**90.63**	25	18.26	0	75.72%
		0.5%	6.28	**66.75**	27.25	0	
		2%	3.09	8.25	**45.5**	0	
		10%	0	0	8.99	**100**	
Corn starch	Recognition	0%	**93.76**	40.93	0	9.13	84.89%
		0.5%	4.69	**59.07**	0	0	
		2%	1.55	0	**95.88**	0	
		10%	0	0	4.12	**90.87**	
	Validation	0%	**87.52**	63.66	0	45.5	54.28%
		0.5%	12.48	**36.34**	0	0	
		2%	0	0	**75**	36.24	
		10%	0	0	25	**18.26**	

**Table 11:** LDA classifications after drift correction, outlier detection and three-fold cross-validation when the adulterant concentration was used as class variable on the e-tongue data of sucrose—and salt—adulterated tomato pastes.

Adulterant	Accuracy		0%	0.5%	1%	5%	Average Classification
Sucrose	Recognition	0%	**96.86**	14.99	9.13	0	80.22%
		0.5%	0	**64.92**	9.13	13.64	
		1%	3.14	10.04	**77.25**	4.5	
		5%	0	10.04	4.5	**81.86**	
	Validation	0%	**93.81**	9.88	18.26	0	56.39%
		0.5%	3.1	**50**	18.26	45.5	
		1%	3.1	20.06	**36.24**	8.99	
		5%	0	20.06	27.25	**45.5**	
Salt	Recognition	0%	**100**	0	0	0	94.49%
		0.5%	0	**91.62**	13.64	0	
		1%	0	8.38	**86.36**	0	
		5%	0	0	0	**100**	
	Validation	0%	**96.9**	0	8.99	0	86.16%
		0.5%	3.1	**75**	18.26	0	
		1%	0	25	**72.75**	0	
		5%	0	0	0	**100**	

**Table 12:** Adulterant concentration prediction with PLSR and “LOOCV” validation on e-tongue sensor signals of authentic and adulterated tomato pastes after drift correction and outlier detection.

Constituent	R^2^_C_	RMSE_C_ (%*w*/*w*)	R^2^_CV_	RMSE_CV_ (%*w*/*w*)	LV	N
Paprika seed	0.9397	0.929	0.9304	0.998	2	58
Corn starch	0.6357	2.215	0.5061	2.574	4	57
Sucrose	0.4925	1.199	0.3305	1.375	4	56
Salt	0.9703	0.307	0.9622	0.346	4	66
Tomato	0.7888	2.625	0.7716	2.730	6	231

R^2^_C_, R^2^_CV_—Coefficient of determination of model building and validation; RMSEC, RMSECV—Root mean square error of calibration and validation; LV—Latent variables; N—Sample count.

## References

[1] Omondi S. (2018). The Most Popular Vegetables in the World. World Atlas.

[2] IndexBox Global Tomato Market 2019—Robust Consumption Growth in China and India Drives the Global Market. https://www.globaltrademag.com/global-tomato-market-2019-robust-consumption-growth-in-china-and-india-drives-the-global-market/.

[3] FAO Stat Crops Tomatoes. http://www.fao.org/faostat/en/#data/QC.

[4] Bertin N., Genard M. (2018). Tomato quality as influenced by preharvest factors. Sci. Hortic..

[5] Fernández-García E., Carvajal-Lérida I., Jarén-Galán M., Garrido-Fernández J., Pérez-Gálvez A., Hornero-Méndez D. (2012). Carotenoids bioavailability from foods: From plant pigments to efficient biological activities. Food Res. Int..

[6] Abdulmalik I.O., Amonye M.C., Ambali A.O., Umeanuka P.O., Mahdi M. (2014). Appropriate technology for tomato powder production. Int. J. Eng. Invent..

[7] FAO, WHO Codex Alimentarius International Food Standards. http://www.fao.org/fao-who-codexalimentarius.

[8] Alimentarius C. (2017). Codex Alimentarius Standard for Processed Tomato Concentrates. Codex Stan.

[9] EC COMMISSION REGULATION (EU) No 1129/2011 of 11 November 2011 Amending Annex II to Regulation (EC) No 1333/2008 of the European Parliament and of the Council by Establishing a Union List of Food Additives. https://eur-lex.europa.eu/legal-content/EN/TXT/HTML/?uri=CELEX:32011R1129&qid=1597164722424&from=HU.

[10] EC REGULATION (EC) No 1333/2008 of the European Parliament and of the Council of 16 December 2008 on Food Additives. https://eur-lex.europa.eu/legal-content/EN/TXT/PDF/?uri=CELEX:32008R1333&qid=1588007840949&from=EN.

[11] Spink J., Moyer D.C. (2011). Defining the Public Health Threat of Food Fraud. J. Food Sci..

[12] Constable K. Food Fraud Risk Information—Tomatoes and Tomato Paste. https://trello.com/c/0BBkItvu/410-tomatoes-and-tomato-paste.

[13] Thakur B.R., Singh R.K., Nelson P.E. (1996). Quality attributes of processed tomato products: A review. Food Rev. Int..

[14] Barringer S.A., Azam A.T.M.S., Heskitt B., Sastry S. (1998). On-line prediction of bostwick consistency from pressure differential in pipe flow for ketchup and related tomato products. J. Food Process. Preserv..

[15] Barringer S.A., Smith J.S., Hui Y.H. (2004). Vegetables: Tomato Processing. Food Processing: Principles and Applications.

[16] Berta M., Wiklund J., Kotzé R., Stading M. (2016). Correlation between in-line measurements of tomato ketchup shear viscosity and extensional viscosity. J. Food Eng..

[17] Arvanitoyannis I.S., Vaitsi O.B. (2007). A review on tomato authenticity: Quality control methods in conjunction with multivariate analysis (chemometrics). Crit. Rev. Food Sci. Nutr..

[18] Callao M.P., Ruisánchez I. (2018). An overview of multivariate qualitative methods for food fraud detection. Food Control.

[19] Osborne B.G. (2006). Near-Infrared Spectroscopy in Food Analysis. Encycl. Anal. Chem..

[20] Davies A.M.C., Dennis C., Grant A., Hall M.N., Robertson A. (1987). Screening of tomato purée for excessive mould content by near infrared spectroscopy: A preliminary evaluation. J. Sci. Food Agric..

[21] Goula A.M., Adamopoulos K.G. (2003). Estimating the composition of tomato juice products by near infrared spectroscopy. J. Near Infrared Spectrosc..

[22] Jha S.N., Matsuoka T. (2004). Non-destructive determination of acid–brix ratio of tomato juice using near infrared spectroscopy. Int. J. Food Sci. Technol..

[23] De Nardo T., Shiroma-Kian C., Halim Y., Francis D., Rodriguez-Saona L.E. (2009). Rapid and simultaneous determination of lycopene and β-carotene contents in tomato juice by infrared spectroscopy. J. Agric. Food Chem..

[24] Deák K., Szigedi T., Pék Z., Baranowski P., Helyes L. (2015). Carotenoid determination in tomato juice using near infrared spectroscopy. Int. Agrophys..

[25] Pedro A.M.K., Ferreira M.M.C. (2005). Nondestructive determination of solids and carotenoids in tomato products by near-infrared spectroscopy and multivariate calibration. Anal. Chem..

[26] Pedro A.M.K., Ferreira M.M.C. (2007). Simultaneously calibrating solids, sugars and acidity of tomato products using PLS2 and NIR spectroscopy. Anal. Chim. Acta.

[27] Sirisomboon P., Tanaka M., Kojima T., Williams P. (2012). Nondestructive estimation of maturity and textural properties on tomato ‘Momotaro’by near infrared spectroscopy. J. Food Eng..

[28] Pasquini C. (2018). Near infrared spectroscopy: A mature analytical technique with new perspectives—A review. Anal. Chim. Acta.

[29] Escuder-Gilabert L., Peris M. (2010). Highlights in recent applications of electronic tongues in food analysis. Anal. Chim. Acta.

[30] Peris M., Escuder-Gilabert L. (2016). Electronic noses and tongues to assess food authenticity and adulteration. Trends Food Sci. Technol..

[31] Liu T., Zhu W., Huang J., Chen H., Nie R., Li C. (2017). Comparison of the nutritional as well as the volatile composition of in-season and off-season Hezuo 903 tomato at red stage. Eur. Food Res. Technol..

[32] Beullens K., Mészáros P., Vermeir S., Kirsanov D., Legin A., Buysens S., Cap N., Nicolaï B.M., Lammertyn J. (2008). Analysis of tomato taste using two types of electronic tongues. Sens. Actuators B Chem..

[33] Xu S., Li J., Baldwin E.A., Rosskopf E., Hong J.C., Bai J. (2017). Differentiation of taste profiles by electronic tongue of full ripe tomato samples from different cultivars and harvest maturities. Proceedings of the Florida State Horticultural Society, Proceedings of the 130th Annual Meeting of the Florida State Horticultural Society, Tampa, FL, USA, 4–6 June 2017.

[34] Xu S., Li J., Baldwin E.A., Plotto A., Rosskopf E., Hong J.C., Bai J. (2018). Electronic tongue discrimination of four tomato cultivars harvested at six maturities and exposed to blanching and refrigeration treatments. Postharvest Biol. Technol..

[35] Beullens K., Kirsanov D., Irudayaraj J., Rudnitskaya A., Legin A., Nicolaï B.M., Lammertyn J. (2006). The electronic tongue and ATR–FTIR for rapid detection of sugars and acids in tomatoes. Sens. Actuators B Chem..

[36] EU COMMISSION REGULATION (EEC) No 1764/86—Laying down minimum quality requirements for products processed from tomatoes under the production aid scheme. https://www.legislation.gov.uk/eur/1986/1764/pdfs/eur_19861764_2001-05-26_en.pdf.

[37] ASTM Standard Test Method for Determining the Consistency of Viscous Liquids Using a Consistometer. https://cds.cern.ch/record/1461360.

[38] McCarthy K.L., Sacher R.F., Garvey T.C. (2008). Relationship between rheological behavior and Bostwick measurement during manufacture of ketchup. J. Texture Stud..

[39] Magwaza L.S., Opara U.L. (2015). Analytical methods for determination of sugars and sweetness of horticultural products—A review. Sci. Hortic..

[40] USDA (1971). Methods of Analysis for Tomato Products.

[41] Alpha M.O.S. (2003). αAstree Electronic Tongue User Manual.

[42] Piepho H.-P. (2004). An algorithm for a letter-based representation of all-pairwise comparisons. J. Comput. Graph. Stat..

[43] Assaad H.I., Zhou L., Carroll R.J., Wu G. (2014). Rapid publication-ready MS-Word tables for one-way ANOVA. Springerplus.

[44] Savitzky A., Golay M.J.E. (1964). Smoothing and differentiation of data by simplified least squares procedures. Anal. Chem..

[45] Dhanoa M.S., Lister S.J., Sanderson R., Barnes R.J. (1994). The link between multiplicative scatter correction (MSC) and standard normal variate (SNV) transformations of NIR spectra. J. Near Infrared Spectrosc..

[46] Kovács Z. (2012). Módszer Elektronikus Nyelvvel Végzett Méréseknél Fellépő Zavaró Hatások Csökkentésére = Method to Decrease the Disturbing Effects Occurring by the Electronic Tongue Measurement.

[47] Kovacs Z., Szöllősi D., Zaukuu J.-L.Z., Bodor Z., Vitális F., Aouadi B., Zsom-Muha V., Gillay Z. (2020). Factors Influencing the Long-Term Stability of Electronic Tongue and Application of Improved Drift Correction Methods. Biosensors.

[48] Kovacs Z., Pollner B. Aquaphotomics-Software R-Package “aquap2”. Proceedings of the Understanding Water in Biology 2nd International Symposium.

[49] Workman J.J. (1996). Interpretive spectroscopy for near infrared. Appl. Spectrosc. Rev..

[50] Galicia-Cabrera R.M. (2007). Tomato Processing. Handbook of Food Products Manufacturing.

[51] Anthon G.E., Barrett D.M. (2010). Changes in tomato paste during storage and the effects of heating on consistency of reconstituted tomato paste. J. Texture Stud..

[52] Sobowale S.S., Olatidoye O.P., Odunmbaku L.A., Raji O.H. (2012). A comparative study on physicochemical and rheological properties of imported tomato paste in Nigeria. Sustain. Agric. Res..

[53] Goodman C.L., Fawcett S., Barringer S.A. (2002). Flavor, Viscosity, and Color Analyses of Hot and Cold Break Tomato Juices. J. Food Sci..

[54] Juszczak L., Oczadły Z., Gałkowska D. (2013). Effect of modified starches on rheological properties of ketchup. Food Bioprocess Technol..

[55] Gujral H.S., Sharma A., Singh N. (2002). Effect of hydrocolloids, storage temperature, and duration on the consistency of tomato ketchup. Int. J. Food Prop..

[56] Diantom A., Curti E., Carini E., Vittadini E. (2017). Effect of added ingredients on water status and physico-chemical properties of tomato sauce. Food Chem..

[57] Xie L., Ying Y. (2009). Use of near-infrared spectroscopy and least-squares support vector machine to determine quality change of tomato juice. J. Zhejiang Univ. Sci. B.

[58] Liu C., Hao G., Su M., Chen Y., Zheng L. (2017). Potential of multispectral imaging combined with chemometric methods for rapid detection of sucrose adulteration in tomato paste. J. Food Eng..

[59] Workman J. (2001). 14. SW-NIR for Organic Composition Analysis. The Handbook of Organic Compounds: NIR, IR, R, and UV-Vis Spectra Featuring Polymers and Surfactants.

[60] Ribeiro J.S., Ferreira M.M.C., Salva T.J.G. (2011). Chemometric models for the quantitative descriptive sensory analysis of Arabica coffee beverages using near infrared spectroscopy. Talanta.

[61] Tsenkova R. (2009). Aquaphotomics: Dynamic spectroscopy of aqueous and biological systems describes peculiarities of water. J. Near Infrared Spectrosc..

[62] Hong X., Wang J. (2015). Use of electronic nose and tongue to track freshness of cherry tomatoes squeezed for juice consumption: Comparison of different sensor fusion approaches. Food Bioprocess Technol..

[63] Hong X., Wang J. (2014). Detection of adulteration in cherry tomato juices based on electronic nose and tongue: Comparison of different data fusion approaches. J. Food Eng..

[64] Winquist F., Krantz-Rülcker C., Lundström I. (2004). Electronic tongues. MRS Bull..

